# Prevalence, Demographic Distribution, and Antimicrobial Resistance Patterns of Extended-Spectrum Beta-Lactamase-Producing Escherichia coli in Urinary Tract Infections: A Cross-Sectional Study in a Tertiary Care Hospital in Manipur, India

**DOI:** 10.7759/cureus.101302

**Published:** 2026-01-11

**Authors:** Bishwabati Devi Yumlembam, Urvashi Chongtham, Manojkumar Singh Rajkumar

**Affiliations:** 1 Microbiology, Shija Academy of Health Sciences, Imphal, IND; 2 Microbiology, Jawaharlal Nehru Institute of Medical Sciences, Imphal, IND

**Keywords:** extended-spectrum beta-lactamase (esbl), multidrug resistant (mdr), northeast india, urinary tract infections, uropathogenic escherichia coli

## Abstract

Background: Urinary tract infections (UTIs) are among the most common bacterial infections globally, with *Escherichia coli* as the predominant uropathogen. The emergence of extended-spectrum beta-lactamase (ESBL)-producing strains has significantly complicated treatment due to widespread multidrug resistance (MDR).

Objectives: This cross-sectional study aimed to determine the prevalence of ESBL-producing *E. coli* in UTIs, describe its demographic distribution, and evaluate antimicrobial susceptibility patterns among isolates from patients at a tertiary care hospital in Imphal, Manipur, India.

Methods: From September 2015 to August 2017, 230 non-repetitive *E. coli* isolates from urine samples of symptomatic patients were collected, and antimicrobial susceptibility testing was done by Kirby-Bauer disk diffusion methodology. Isolates were then screened for ESBL production using Clinical and Laboratory Standards Institute (CLSI) guidelines. Screening employed disk diffusion with indicator cephalosporins, and confirmation for ESBL production was done via the combined disk test (CDT) (ceftazidime ± clavulanic acid).

Results: Of the 230 isolates, 171 (74.3%) were screened positive and 97 (56.7%) were confirmed as ESBL producers by CDT. Female patients predominated with 185 (80.4%) cases; the male-to-female ratio was 1:4.1, with the highest prevalence in the 16-25 years age group, accounting for 44 (19.1%) cases. Community-acquired isolates represented 172 (74.8%) cases, with 68 (70.1%) showing ESBL positivity versus 29 (29.9%) in inpatient isolates. All isolates retained 100% sensitivity to imipenem. ESBL producers exhibited complete resistance to cefpodoxime, 7 (7.2%) to gentamicin, and high co-resistance to ciprofloxacin. Non-producers showed significantly lower resistance rates (p < 0.05).

Conclusions: This study demonstrates a high prevalence of ESBL-producing *E. coli* in both community and hospital settings in Manipur, underscoring the urgent need for routine screening, enhanced antimicrobial stewardship, ongoing surveillance, and evidence-based empirical therapy guidelines to curb escalating resistance and improve patient outcomes.

## Introduction

Urinary tract infections (UTIs) represent a significant global health burden, causing millions of outpatient visits and hospitalizations annually. In the United States alone, community-acquired UTIs cost approximately $1.6 billion yearly [1]. Female individuals are disproportionately affected, with nearly half experiencing at least one episode in their lifetime, primarily due to anatomical factors [2]. *Escherichia coli* remains the most common etiological agent, but rising antimicrobial resistance (AMR), particularly through extended-spectrum beta-lactamases (ESBLs), poses a major challenge [3]. ESBLs hydrolyze broad-spectrum cephalosporins and monobactams, conferring multidrug resistance (MDR) and limiting therapeutic options [4].

In India, ESBL prevalence among Enterobacteriaceae varies from 20% to 71%, with limited data from regions like Manipur [5]. This study addresses this gap by investigating the prevalence, demographic distribution, and antibiotic susceptibility patterns of ESBL-producing uropathogenic *E. coli* in a tertiary care hospital in Imphal, Manipur. Understanding local patterns is crucial for guiding empirical therapy and formulating antibiotic policies to mitigate resistance [6].

## Materials and methods

This descriptive cross-sectional study was conducted in the Department of Microbiology, Jawaharlal Nehru Institute of Medical Sciences, Imphal, from September 2015 to August 2017. Ethical approval was obtained from the institutional ethics committee, and informed consent was secured from participants or guardians.

Inclusion criteria encompassed non-repetitive urine samples from patients aged 5-80 years with UTI symptoms (e.g., dysuria, frequency) from the outpatient departments (OPD) and inpatient departments (IPD). Clean-catch midstream urine (10-15 mL) was collected in sterile containers. Exclusion criteria included children under five years, catheterized patients, repeated samples, mixed growth (>3 organisms), and non-*E. coli* isolates.

Sample size was calculated as 230 using the standard formula: n = (z^2^pq)/d^2^, where z=1.96, p=18.5% from prior studies, q=81.5%, and d=5%.

Samples underwent microscopic examination for pus cells and bacteria, followed by semi-quantitative culture on cystine lactose electrolyte deficient (CLED), MacConkey, and blood agar. Significant bacteriuria was defined as ≥10^5^ CFU/mL. *E. coli *identification involved colony morphology, Gram staining, motility, and biochemical tests (catalase, oxidase, indole, methyl red, Voges-Proskauer, citrate, urease, triple sugar iron (TSI), sugar fermentation, nitrate reduction, oxidation-fermentation, decarboxylase).

Antimicrobial susceptibility was tested on Mueller-Hinton agar using the Kirby-Bauer disk diffusion method as per Clinical and Laboratory Standards Institute (CLSI) 2014 guidelines [7].

Screening of ESBL production

ESBL screening used disk diffusion with ceftazidime (≤22 mm), ceftriaxone (≤25 mm), cefotaxime (≤27 mm), cefpodoxime (≤17 mm), and aztreonam (≤27 mm) zones, indicating positives. Confirmation primarily used the combined disk test (CDT) (ceftazidime ± clavulanic acid; ≥5 mm zone increase for positives). *E. coli *ATCC 25922 served as the control.

Confirmation of ESBL production

*E. coli* isolates identified as potential ESBL producers were listed for confirmation of ESBL production and were further subjected to CDT. This test requires the use of a third-generation cephalosporin antibiotic disk alone in combination with clavulanic acid. For the purpose of this study, disks containing 30 mcg of ceftazidime and disks containing a combination of ceftazidime plus 10 μg of clavulanic acid (HiMedia, Mumbai, India) were placed independently, 30 mm apart, on a lawn culture of the test isolate on a Mueller-Hinton agar plate and incubated overnight at 37°C. The difference in zone diameters with and without clavulanic acid was measured.

Isolates were considered ESBL positive if the inhibition zone measured around the combination disks was found to be at least 5 mm larger than that of the corresponding cephalosporin disk.

Data were analyzed descriptively, with chi-square tests for associations (p<0.05 was considered significant).

## Results

Of the 230 isolates, the mean patient age was 36.7 ± 19.6 years (range 6-87, median 35), with the highest cases in the 16-25 years age group (44 (19.1%)), followed by 26-35 years age group (43 (18.7%)) and 36-45 years age group (40 (17.4%)). Female patients comprised 185 (80.4%), with a male-to-female ratio of 1:4.1. OPD patients dominated with a total of 172 (74.8%) cases, while maximum IPD samples were received from gynecology/obstetrics.

The antibiotic susceptibility pattern of *E. coli* isolates was performed by the Kirby-Bauer disk diffusion method on cefpodoxime, ceftazidime, cefotaxime, aztreonam, ceftriaxone, amoxycillin/clavulanic acid, ceftazidime/clavulanic acid, nitrofurantoin, gentamicin, imipenem, cefepime, and ciprofloxacin. The overall antibiotic susceptibility pattern of all 230 *E. coli* isolates, determined by Kirby-Bauer disk diffusion, is presented in Table 1.

**Table 1:** Distribution of antibiotics by sensitivity pattern CPD: cefpodoxime; CAZ: ceftazidime; CTX: cefotaxime; AZ: aztreonam; CEF: ceftriaxone; AMC: amoxycillin/clavulanic acid; CAZ/CA: ceftazidime/clavulanic acid; NIT: nitrofurantoin; GEN: gentamicin; IPM: imipenem; CPM: cefepime; CIP: ciprofloxacin

Antibiotics	Sensitive, n (%)	Resistant, n (%)	Intermediate, n (%)
CPD	79 (34.3)	130 (56.5)	21 (9.1)
CAZ	86 (37.4)	98 (42.6)	46 (20.0)
CTX	77 (33.5)	114 (49.6)	39 (17.0)
AZ	94 (40.9)	124 (53.9)	12 (5.2)
CEF	72 (31.3)	122 (53.0)	36 (15.7)
AMC	144 (49.6)	99 (43.0)	17 (7.4)
CAZ/CA	129 (56.1)	87 (37.8)	14 (6.1)
NIT	193 (83.9)	9 (3.9)	28 (12.2)
GEN	161 (70.0)	23 (10.0)	46 (20.0)
IPM	230 (100.0)	0 (0.0)	0 (0.0)
CPM	69 (30.0)	143 (62.2)	18 (7.8)
CIP	145 (63.0)	45 (19.6)	40 (17.4)

All 230 isolates were subjected to ESBL screening, with results shown in Table 2. Of these, 171 (74.3%) screened positive and were further subjected to confirmatory testing by CDT.

**Table 2:** Distribution of respondents by screening test and confirmatory test for ESBL production CDT: combined disk test; ESBL: extended-spectrum beta-lactamase

Result	Number	Percentage
Screening test (N=230)		
Positive	171	74.3
Negative	59	25.7
Confirmatory test by CDT (N=171)		
Positive	97	56.7
Negative	74	43.3

The antibiotic sensitivity patterns of all ESBL-producing and non-ESBL-producing isolates were compared, as shown in Table 3. Notable findings included 100% resistance to cefpodoxime among ESBL producers, significantly higher resistance to most third-generation cephalosporins, and retained 100% sensitivity to imipenem in both groups. In the ESBL-producing group, the isolates were all resistant to cefpodoxime, whereas in the non-ESBL-producing group, 33 (44.6%) isolates were resistant to cefpodoxime (Table 3). The finding was found to be statistically significant (p<0.05).

**Table 3:** Antibiotic sensitivity pattern of ESBL-producing and non-ESBL-producing Escherichia coli CPD: cefpodoxime; CAZ: ceftazidime; CTX: cefotaxime; AZ: aztreonam; CEF: ceftriaxone; AMC: amoxycillin/clavulanic acid; CAZ/CA: ceftazidime/clavulanic acid; NIT: nitrofurantoin; GEN: gentamicin; IPM: imipenem; CPM: cefepime; CIP: ciprofloxacin; ESBL: extended-spectrum beta-lactamase

Antibiotics	Category	ESBL producing, n (%)	Non-ESBL producing, n (%)	Total, n (%)	Chi-square value	df	p-value
CPD	Sensitive	0 (0.0)	21 (28.4)	21 (12.3)	70.69	2	<0.001
	Resistant	97 (100.0)	33 (44.6)	130 (76.0)			
	Intermediate	0 (0.0)	20 (27.0)	20 (11.7)			
CAZ	Sensitive	6 (6.2)	21 (28.4)	27 (15.8)	118	2	<0.001
	Resistant	90 (92.8)	8 (10.8)	98 (57.3)			
	Intermediate	1 (1.0)	45 (60.8)	46 (26.9)			
CTX	Sensitive	4 (4.1)	15 (20.3)	19 (11.1)	18.5	2	<0.001
	Resistant	77 (79.4)	37 (50.0)	114 (66.7)			
	Intermediate	16 (16.5)	22 (29.7)	38 (22.2)			
AZ	Sensitive	8 (8.2)	27 (36.5)	35 (20.5)	23.5	2	<0.001
	Resistant	84 (86.6)	40 (54.1)	124 (72.5)			
	Intermediate	5 (5.2)	7 (9.5)	12 (7.0)			
CEF	Sensitive	7 (7.2)	8 (10.8)	15 (8.8)	10.7	2	0.005
	Resistant	78 (80.4)	43 (58.1)	121 (70.8)			
	Intermediate	12 (12.4)	23 (31.1)	35 (20.5)			
AMC	Sensitive	82 (84.5)	31 (41.9)	113 (66.1)	44.3	2	<0.001
	Resistant	7 (7.2)	39 (52.7)	46 (26.9)			
	Intermediate	8 (8.2)	4 (5.4)	12 (7.0)			
CAZ/CA	Sensitive	96 (99.0)	33 (44.6)	129 (75.4)	77	2	<0.001
	Resistant	1 (1.0)	28 (37.8)	29 (17.0)			
	Intermediate	0 (0.0)	13 (17.6)	13 (7.6)			
NIT	Sensitive	83 (85.6)	60 (81.1)	143 (83.6)	–	–	–
	Resistant	4 (4.1)	2 (2.7)	6 (3.5)			
	Intermediate	10 (10.3)	12 (16.2)	22 (12.9)			
GEN	Sensitive	71 (73.2)	51 (68.9)	122 (71.3)	1.8	2	0.392
	Resistant	7 (7.2)	10 (13.5)	17 (9.9)			
	Intermediate	19 (19.6)	13 (17.6)	32 (18.7)			
IPM	Sensitive	97 (100.0)	74 (100.0)	171 (100.0)	–	–	–
	Resistant	0 (0.0)	0 (0.0)	0 (0.0)			
	Intermediate	0 (0.0)	0 (0.0)	0 (0.0)			
CPM	Sensitive	16 (16.5)	19 (25.7)	35 (20.5)	5.7	2	0.055
	Resistant	76 (78.4)	46 (62.2)	122 (71.3)			
	Intermediate	5 (5.2)	9 (12.2)	14 (8.2)			
CIP	Sensitive	60 (61.9)	40 (54.1)	100 (58.5)	2.1	2	0.344
	Resistant	23 (23.7)	17 (23.0)	40 (23.4)			
	Intermediate	14 (14.4)	17 (23.0)	31 (18.1)			
Total		97 (100.0)	74 (100.0)	171 (100.0)	–	–	–

The majority of ESBL-producing uropathogenic *E. coli *strains were found in patients of the reproductive age group (26-45 years), accounting for 83 (36.1%) cases. Female patients showed much higher cases of ESBL production compared to male patients. The maximum production of ESBL was seen in OPD with 68 (70.1%) cases. 

All isolates showed 100% sensitivity to imipenem. ESBL producers showed 100% resistance to cefpodoxime, high resistance to ceftazidime, cefotaxime, ceftriaxone, and aztreonam. Non-ESBL isolates had lower resistance (e.g., 33 (44.6%) to cefpodoxime; p<0.05). Gentamicin sensitivity was 17 (23%) in the ESBL group and 51 (68.9%) in the non-ESBL group. Nitrofurantoin sensitivity was 60 (81.1%) in the non-ESBL group and 83 (85.6%) in the ESBL group.

## Discussion

Cephalosporins, particularly third-generation ones, have been widely used for decades to treat infections caused by Gram-negative bacteria. However, their overuse has led to the emergence of ESBLs, which render these antibiotics ineffective [6]. Comparing ESBL prevalence across studies is challenging due to variations in study designs. Many studies, including the present one, confirm that UTIs are more common in female patients, especially in the reproductive age group (25-45 years), with the highest ESBL production of 83 (36.1%) observed in this demographic [2,8,9]. This increased susceptibility in female patients is attributed to their shorter urethra, facilitating easier bacterial access to the bladder [2,9].

Originally considered a hospital-acquired problem, ESBL production is now prevalent in community-acquired isolates, particularly *E. coli*. In this study, 68 (70.1%) of *E. coli *isolates were community-acquired (from OPDs), while 29 (29.9%) were seen in inpatients. Community-acquired ESBL-producing isolates showed greater resistance to third-generation cephalosporins compared to inpatients, consistent with findings from other studies [10,11]. This high resistance is linked to the irrational and rampant use of third-generation cephalosporins in both community and hospital settings, lack of antibiotic policies, and factors such as invasive procedures and high antibiotic pressure, which promote mutations and the emergence of resistant strains [6].

ESBL prevalence rates in India vary widely. The current study reported a prevalence of 97 (56.7%), which aligns closely with several other Indian studies reporting rates between 51.4% and 70% [3,5,11]. The relatively high rate in this study may be attributed to factors such as indwelling catheters, invasive procedures, severity of illness, and excessive cephalosporin use. This rising incidence among clinical isolates raises concerns about potential treatment failures due to resistant organisms. One study reported a notably higher ESBL production of 97%, likely because it focused exclusively on third-generation cephalosporin-resistant organisms [11].

Recent multicentric and regional data indicate persistently high ESBL rates exceeding 50-66% nationwide and in various regions, with community-acquired cases driving the surge, mirroring our findings and underscoring unchecked selection pressure from third-generation cephalosporins [11,12].

ESBL-producing organisms often exhibit co-resistance to other antibiotic classes, particularly fluoroquinolones and aminoglycosides, due to resistance genes being located on the same plasmids [10]. In this study, only 17 (23%) isolates were susceptible to gentamicin, contrasting with higher resistance rates reported in other regional studies [11]. These variations may stem from differing levels of aminoglycoside use and selection pressure.

Carbapenems, such as imipenem, remain highly effective against most ESBL-producing pathogens, with 100% sensitivity observed in this study. However, emerging carbapenem resistance (e.g., via enzymes like NDM-1) is a growing concern worldwide, limiting therapeutic options [13]. In such cases, colistin may be required despite its significant side effects, underscoring the urgent need for antibiotic stewardship to prevent further escalation of resistance [6].

Prevalence and regional context

The 97 (56.7%) prevalence of ESBL production among uropathogenic *E. coli *in Manipur aligns with historical and recent Indian rates of 53-70% but reveals an alarming persistence in an understudied northeast region [11]. The 171 (74.3%) screening positivity rate demands immediate integration of routine phenotypic confirmation in local laboratories to avert empirical therapy failures.

Demographic patterns and community acquisition

Female predominance of 185 (80.4%) cases and reproductive-age peaking reflect classic anatomical and hormonal vulnerabilities [2,8,9]. Critically, the dominance of community-acquired isolates of 68 (70.1%) ESBL-positive in outpatients blurs traditional nosocomial boundaries, echoing South Asian trends where irrational over-the-counter use fuels plasmid-mediated dissemination beyond hospitals [10,11].

Antimicrobial susceptibility and resistance mechanisms

Imipenem's retained 100% efficacy offers a reliable reserve, yet emerging carbapenemase threats loom [13]. High co-resistance, e.g., 7 (7.2%) to gentamicin, highlights multidrug plasmids encoding efflux pumps and modified targets, severely limiting alternatives like nitrofurantoin. These patterns exemplify intense selective pressure from antibiotic misuse, amplifying morbidity amid India's escalating AMR burden.

Limitations and strategic implications

Single-center scope and absent genotypic analysis (e.g., CTX-M prevalence) limit broader generalizations. Multicenter One Health studies incorporating molecular typing are essential [13]. For Manipur and northeast India, these results compel targeted stewardship: restricting cephalosporin access, enhancing surveillance, and educating clinicians to preserve carbapenems. Proactive measures could stem resistance escalation, safeguarding UTI management in vulnerable populations.

## Conclusions

The high prevalence of ESBL-producing *E. coli* in UTIs in Manipur shows that resistance is now a serious problem in both community and hospital settings. Clinicians must follow antibiotic stewardship practices and avoid unnecessary use of cephalosporins to slow down the spread of resistance. Hospitals and public health authorities should start routine screening for these resistant strains and keep monitoring local patterns. These steps will help preserve effective treatment options and improve outcomes for patients with UTIs.
